# Portrait of Italian Cardio-Oncology: Results of a Nationwide Associazione Nazionale Medici Cardiologi Ospedalieri (ANMCO) Survey

**DOI:** 10.3389/fcvm.2021.677544

**Published:** 2021-06-16

**Authors:** Maria Laura Canale, Fabio Turazza, Chiara Lestuzzi, Iris Parrini, Andrea Camerini, Giulia Russo, Furio Colivicchi, Domenico Gabrielli, Michele Massimo Gulizia, Stefano Oliva, Luigi Tarantini, Nicola Maurea, Luigi Rigacci, Sandro Petrolati, Giancarlo Casolo, Irma Bisceglia

**Affiliations:** ^1^Division of Cardiology, Azienda USL Toscana Nord-Ovest, Versilia Hospital, Lido di Camaiore, Italy; ^2^Cardiology Unit, Fondazione IRCCS Istituto Nazionale dei Tumori, Milan, Italy; ^3^Cardiology Unit, Oncology Department, CRO National Cancer Institute, Aviano, Italy; ^4^Divisione di Cardiologia, Ospedale Mauriziano, Turin, Italy; ^5^Medical Oncology, Azienda USL Toscana Nord-Ovest, Versilia Hospital, Lido di Camaiore, Italy; ^6^SC Centro Cardiovascolare Ospedale Maggiore, Trieste, Italy; ^7^Division of Cardiology, San Filippo Neri Hospital, ASL Roma 1, Rome, Italy; ^8^Division of Cardiology, Azienda Ospedaliera San Camillo-Forlanini, Rome, Italy; ^9^Division of Cardiology, Garibaldi-Nesima Hospital, Catania, Italy; ^10^UOSD Cardiologia di Interesse Oncologico - IRCCS Istituto Tumori “GIOVANNI Paolo II” Bari, Bari, Italy; ^11^Division of Cardiology, Arcispedale S. Maria Nuova, Azienda USL – IRCCS di Reggio-Emilia, Reggio-Emilia, Italy; ^12^S.C. Cardiologia, Istituto Nazionale Tumori, IRCCS Fondazione G. Pascale, Napoli, Italy; ^13^UOC Ematologia, Azienda Ospedaliera San Camillo-Forlanini, Rome, Italy; ^14^Servizi Cardiologici Integrati, Azienda Ospedaliera San Camillo-Forlanini, Rome, Italy

**Keywords:** cardio-oncology, anthracyclines, trastuzumab, global longitudinal strain, cardiac biomarker, healthcare

## Abstract

**Aims:** Cardio-oncology has achieved a pivotal role in science, but real world data on its clinical impact are still limited.

**Methods:** A questionnaire was sent out to all cardio-oncology services across Italy (*n* = 120). The questionnaire was made up of 28 questions divided into four blocks: (A) general information on hospitals and service, (B) the inner organization of cardio-oncology and its relationships with out-of-hospital cardiologists and general practitioners, (C) educational needs and referral guidelines, and (D) activities/specific workload.

**Results:** Ninety-six out of 120 (80%) completed the questionnaire; 9.4% were cancer centers while 90.6% were general hospitals. A cardio-oncology team was present in 56% of the cancer centers and in 20% only of general hospitals, and a cardio-oncology pathway was active in 55% of cancer centers and in just 14% of the general hospitals. Relationships with out-of-hospital cardiologists and general practitioners were lacking. The guidelines of reference were ESC and ANMCO/AIOM. Patients receiving anthracycline chemotherapy underwent scheduled monitoring by means of echocardiography in 58% of cases. Routine use of cardiac damage biomarkers was overall low, ranging from 22 to 33% while the use of global longitudinal strain reached 44%.

**Conclusions:** Italian cardio-oncology showed a growing influence on clinical practice but still has room for improvement. Cardio-oncology teams are still scarce, and the application of dedicated paths is poor. The need for specific training has been highlighted.

## Introduction

After a long period of being overlooked, cardio-oncology (CO) is now playing a major role in the clinical scenario of both cardiology and oncology. The leading cardiology and medical oncology organizations have recently released guidelines and recommendations (1–3) on the subject. In addition, they have provided advice on how to set up a CO program (4, 5). Accordingly, an increasing number of national cardiology societies have published CO reports (6–8), and the number of Internet searches on CO related topics has increased (9). Recommendations and guidelines are fundamental tools for cardiologists who intend to provide the best care to cancer patients and fulfill the scientific need for CO. CO being a relatively new discipline, guideline indications do not directly or automatically apply to clinical daily practice. Recently, a survey on cardiac imaging in CO highlighted considerable gaps between guidelines and everyday clinical practice (10).

The lack of specific clinical pathways and of clinicians' confidence makes the widespread application of the guidelines slower and more difficult. Moreover, the quantity of real world data on the clinical application of these recommendations is limited. On these grounds, we conducted an Italian nationwide survey on the behalf of the Associazione Nazionale Medici Cardiologi Ospedalieri (ANMCO) to paint a detailed picture of the daily behaviors of professionals dealing with cardiac care in cancer patients.

## Methods and Materials

On July 18th, 2019, a CO questionnaire was uploaded on the ANMCO website on the behalf of Cardio-Oncology Task Force. Deadline for submission was set on January 22th, 2020. An invitation email was sent out to the regional ANMCO presidents, regional CO delegates (identified by the presidents), and the referral cardiologists of each CO service all across Italy. A complete list of CO facilities was available based on the results of the two previous ANMCO surveys in 2017 and 2019. The ANMCO database of CO services was the only one available, and it covers the national territory with few exceptions, so survey results could offer a reliable picture of real-world Italian CO practice. Before compilation, each participant signed up and clearly identified the center for which he or she worked. Completed questionnaires were double-checked to avoid duplicates from the same center.

The aims of the survey were to characterize the activity of CO services across Italy; to explore their network of cancer-treating physicians, general practitioners and out-of-hospital cardiologists; and to analyze their educational needs.

The questionnaire was composed of 28 single or multiple-choice questions divided into four functional domains: (A) general information on hospitals and service (questions 1–4); (B) the inner organization of cardio-oncology and its relationships with out-of-hospital cardiologists and general practitioners (questions 5–13); (C) educational needs and referral guidelines (Questions 14–16); and (D) activity (questions 17–28).

The first block of questions aimed to analyze the types of hospitals in which the cardio-oncology services operate and the inner organization of the oncology referral department. Questions about service-related cancer patients (type of cancer and provenance) were also a part of this block. The second part inquired about the organization of the cardio-oncology service. It was asked about the frequency and the modalities (direct case-by-case phone calls, written advice, etc.) of the relationships between clinicians and general practitioners of the surrounding area. Questions regarding the relationship with out-of-hospital cardiologists were also included in this block while the relationships with out-of-hospital oncologists and hematologists were not part of the survey. The third part explored the educational needs of cardio-oncology service staff; both nurses and physicians were questioned about their interest on specific (additional) training by means of an ANMCO educational focused activity. Moreover, they were asked about their referral guidelines.

The fourth one focused on CO workload. Clinicians were asked about the categories of patients receiving potentially cardio-toxic drugs who underwent regular cardiac follow-up; how they performed risk assessment for cardiac toxicity of anthracyclines; the timeline of cardiological evaluation of patients receiving anthracyclines or trastuzumab; and how they managed new cardiac toxicity and/or new drugs. Information about the use of cardiac damage biomarkers and global longitudinal strain (GLS) in early detection of left-ventricular ejection fraction decrease were also part of this section.

The survey did not require approval by the Local Ethical Committee because it is based on physicians' opinions and administrative data only, without direct patient data collection.

Because of the descriptive aim of the survey, no formal statistical design was set up. Data are presented as percentages of the whole number of answers received for each single or multiple question. Multiple answers were possible for some questions.

## Results

**Figure 1:**
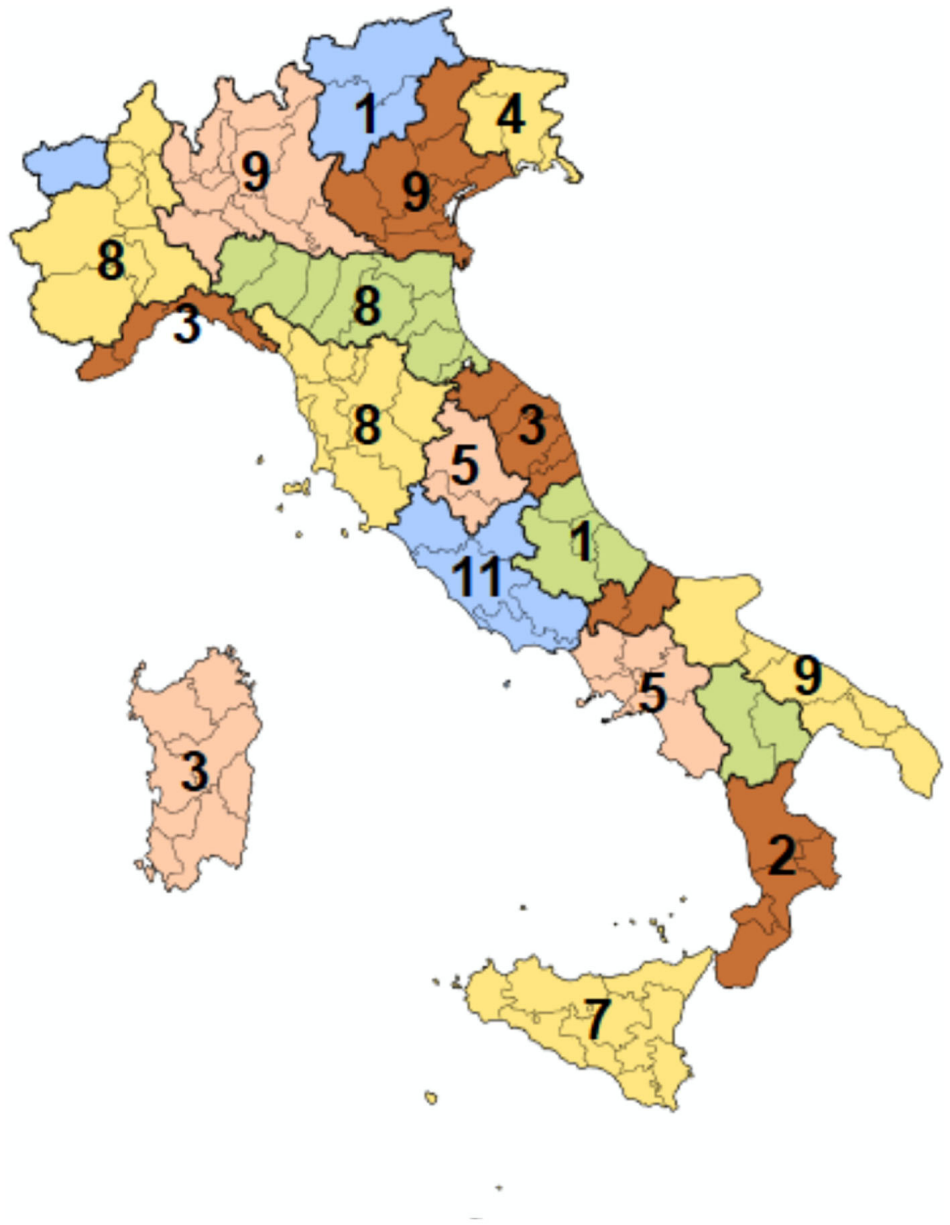
Geographical distribution by region of centers included in the survey (*n* = 96).

On the deadline, 80% of centers (96 out of 120) completed the online questionnaire and were therefore included in this report. The geographical distribution of the centers that completed the survey is shown in Figure 1.

### Domain A: General Information on Hospitals and Service

Nine out of 96 hospitals (9.4%) were cancer centers, while in the large majority of cases (87 out of 96, 90.6%) CO operated in a general hospital. Twelve out of 87 (13.8%) hospitals in the general hospital category could be classified as tertiary referral establishments. At a glance, an uneven distribution appears with a slight prevalence of participating centers of the northern and central regions of Italy (34 in the North vs. 36 in the central and 26 in the southern regions) and a paired, rather than homogenous, availability of CO services.

**Figure 2:**
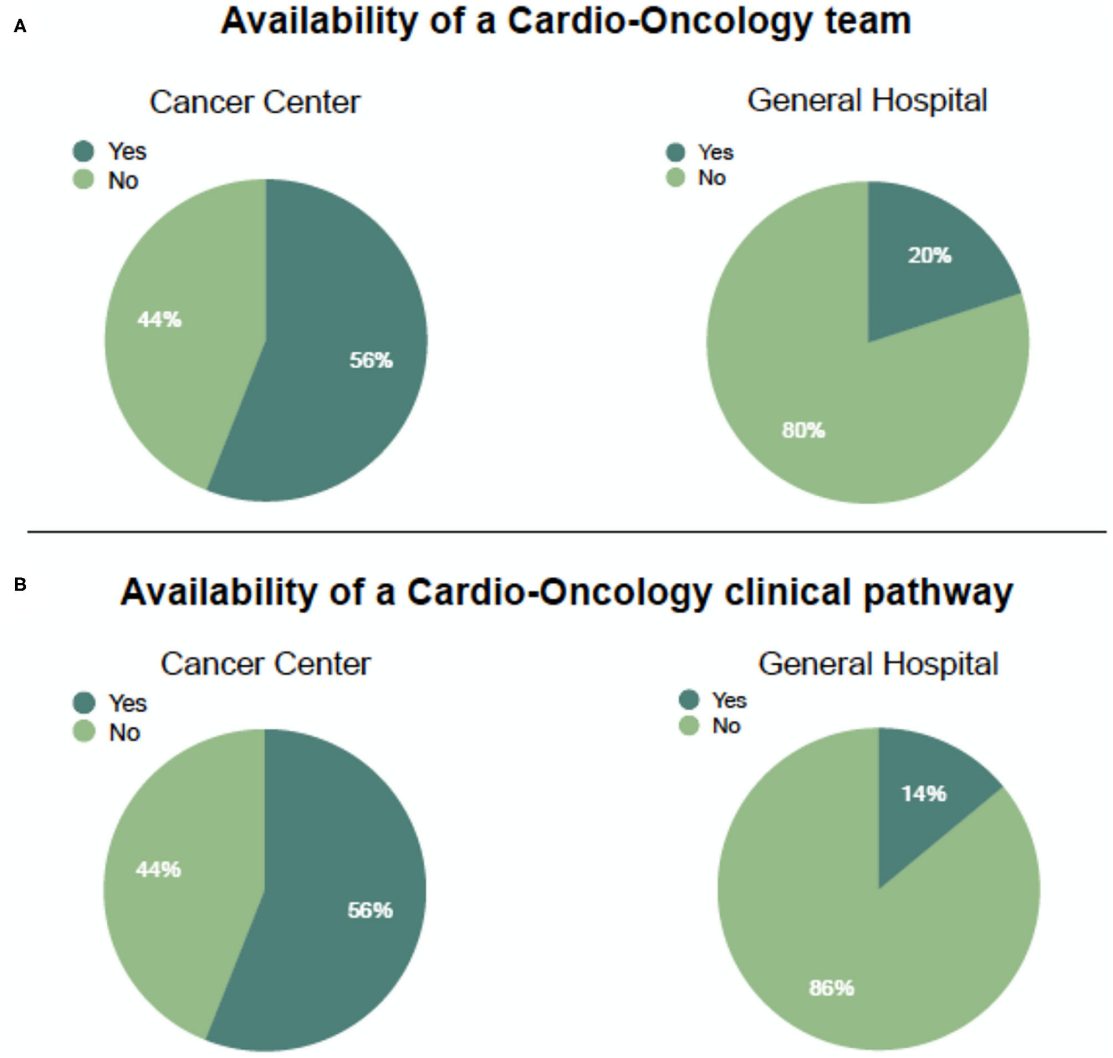
Availability of a cardio-oncology team **(A)** and a dedicated clinical pathway **(B)** in Italian centers having completed the survey, divided by hospital type.

As expected, all cancer centers took care of a wide range of cancer patients, including rare ones, but also general hospitals with a cardio-oncology service dealt with more than three cancer types in 77% of cases. Overall, the more frequent cancer type requiring a CO consultation was breast cancer, followed by lung cancer and gastro-intestinal cancer. The differences in CO teams between cancer centers and general hospitals were remarkable. While in five out of nine (56%) cancer centers an official team or a pool of dedicated cardio-oncologists was available, this percentage dropped to 20% (18 out of 87) in general hospitals, leading to an overall percentage of 24% (Figure 2, top panel).

### Domain B: Inner Organization of Cardio-Oncology and Relationships With Out-of-Hospital Cardiologists and General Practitioners

The differences between cancer centers and general hospitals surfaced when cardiologists were asked about their relationship with oncologists. A shared clinical CO protocol was active (even if with different modalities including multidisciplinary meetings only) in 55% of cancer centers while only 14% of CO services in general hospitals had an organized rule-based clinical pathway (Figure 2, bottom panel). Overall, centers suffered because of the absence of nurses in the team; only in 27% of responding hospitals was a nurse always on the team. Cooperation with out-of-hospital cardiology services was lacking in both settings. Fifty-eight out of 87 (66.6%) general hospitals and four out of nine (44%) cancer centers did not share information on patients with territorial cardiologists in a planned way. In some cases (22%), information is sent out from cancer centers to external specialists, due to the distance between the patient's home and the center itself. This percentage drops to 10% for general hospitals. In both facilities, information with out-of-hospital cardiologists is shared on a single-case base, limited just to complex ones. When asked about relationships with general practitioners, respondents reported a tighter bond. Communication was frequent in only 9% and occasional in 57% of cases leading to an overall percentage of collaboration of 68%. Information was shared mainly by means of the discharge summary (67%) or by a phone call (41%).

### Domain C: Educational Needs and Referral Guidelines

Vocational training paths still represent an unmet need of cardio-oncology staff. An analysis of answers highlighted the willingness to participate in focused CO learning programs in 81.2% (78 out of 96) of nurses and in 91.6% (88 out of 96) of clinicians. The reasons for interest in CO training programs were the need for skill improvement (78%) in hospitals where a CO program is already active and initial training in centers lacking a CO team (14%). The guidelines of reference in clinical practice for Italian cardio-oncologists were those of ESC in 64.6% (62 out of 96) and the consensus of ANMCO and AIOM with a similar percentage, 64.6%. American guidelines from ASCO (20%) or cancer-site specific guidelines (21%) were less followed.

### Domain D: Activity

The majority of centers (60%) offers a dedicated path to cancer patients for all cardiotoxic drugs with anthacyclines (52%), trastuzumab (51%), immune check-point inhibitors (ICIs) (31%), and tyrosine kinase inhibitors (TKIs) (31%) being the most frequently used drugs. The sound criteria in the literature for the cardiac toxicity of anthracyclines were known and applied constantly (82%). The presence of anthracyclines cardiac toxicity risk factors was checked on an equal basis by cardiologists (42%) or oncologists (43%) but always according to a predefined checklist. The surveillance of cancer patients undergoing treatment with anthracyclines seemed to be quite well-established; overall, 58% of centers routinely performed a scheduled monitoring with echocardiography before and after treatment, with 56% increased frequency in high-risk patients. An end-of-treatment echocardiogram was performed in 68% of cases and 45% performed an additional echocardiogram after 1 year (Table 1, upper panel). Trastuzumab treatment was paired with close monitoring; the 3-month control schedule is met in 71% of general hospitals and 89% of cancer centers (Table 1, bottom panel).

**Table 1:** Frequency of routine cardiac evaluations in cancer patients receiving anthracyclines (upper panel) or trastuzumab (lower panel) by center type.

	**General hospital**	**Cancer center**
Before and after treatment (*n*/%)	53/87 (60%)	5/9 (55%)
1-year after completion (*n*/%)	39/87 (45%)	4/9 (44%)
3-months schedule	62/87 (71%)	8/9 (89%)

A low rate of routine utilization of cardiac biomarkers was observed in patients receiving anthracyclines; B-type natriuretic peptide (BNP) and/or NT-pro-BNP alone were routinely used in only 2% of general hospitals, and this percentage slightly rose up to 9% for troponins with a prevalence of troponin T over troponin I. The routine coupled use of these biomarkers reached a percentage of 22% in both general hospitals and cancer centers. Overall, 32% of general hospitals and 22% of cancer centers routinely use any biomarkers to monitor cardiac toxicity of anthracyclines (Table 2, upper panel). The use of cardiac biomarkers was slightly more frequent in specific populations as patients at high risk for cardiac toxicity or to those with a suspect of toxicity.

**Table 2:** Use of cardiac biomarkers (upper panel) and global longitudinal strain (lower panel) in the routine monitoring of cancer patients receiving anthracyclines by center type.

	**General hospital**	**Cancer center**
Troponin T or I (*n*/%)	7/87 (8%)	0/9 (0%)
BNP or NT-pro-BNP (*n*/%)	2/87 (2%)	0/9 (0%)
Troponin plus BNP or NT-pro-BNP (*n*/%)	19/87 (22%)	2/9 (22%)
Global longitudinal strain^*^	37/87 (43%)	4/9 (44%)

*BNP, B-type natriuretic peptide*;

* *Taking together the answers “always” and “depending on the operator” (see text for details)*.

Data on the use of GLS were more reassuring. When we take the answers “always” and “depending on the operator” together, the global percentage of use was 43% and 44% in general hospitals and cancer centers, respectively (Table 2, bottom panel).

Patients with a history of coronary artery disease and a planned fluoropyrimide-based treatment underwent a pretreatment exercise stress test or imaging stress tests in 43% of cases, and a similar percentage is subjected to ECG-monitoring during the initials days of therapy. Thrombosis in cancer patients is mainly managed by cardiologists (53%), followed by oncologists (32%) and internal medicine specialists (26%); interestingly, a multidisciplinary approach is reported in 24%.

## Discussion

Over the past decade CO has played a major role in the management of cancer patients in Italy. While its role from a scientific and educational point of view has been widely recognized due to the tireless work of scientific societies, the real world impact on daily clinical practice is still too limited. A previous report (11) showed that the percentage of hospitals offering a dedicated CO service was 20% in Tuscany, and the overall national percentage was observed to be slightly higher (24%), and almost half of cancer centers do not have a CO team. As a matter of fact, CO services are still underrepresented and show regional disparities.

Issues on CO availability are not only limited to geographical distribution. The geographical differences we observed, with a slightly higher prevalence of centers with a CO service in the northern and central Italian regions, are not just the natural consequences of the distribution of cancer centers, which are mainly located in these regions.

Clinical CO pathways are lacking in the majority of hospitals, and cooperation among physicians is mainly on a single-case base. Similarly, multidisciplinary meetings are not tightly scheduled. In a minority of centers only one nurse is allocated to the CO team. The relationship among cancer centers, general hospitals, and out-of-hospital facilities was a major focus of the survey. A previous ANMCO report highlighted the need for a multidisciplinary inter-hospital network in order to offer full cardiological assistance to cancer patients (12). Survey results clearly showed that cooperation with out-of-hospital cardiologists or general practitioners, regardless of hospital characteristics, is far from effective. The difference between the rising interest in CO and its low availability observed in Italy was also outlined in a recent report from the ESC Cardio-Oncology council (4).

The educational work carried out by scientific societies (ESC, ESMO, ASCO, ANMCO/AIOM) to create specific CO guidelines has achieved consistent results. All survey participants declared that they were aware of the existence of specific guidelines, with ESC and ANMCO being the best known and applied. This is undoubtedly related to the increasing attention of both cardiologists and oncologists to CO issues over the last few years and the commitment of scientific societies to seek clinicians' attention with dedicated activities and focused guidelines. The need for specific training is strongly felt by cardiologists and nurses as well as by the wide majority of centers, which are interested in CO courses. Probably, from a national perspective, the classic educational activities (i.e., focused events or dedicated sessions within major congresses) should be coupled with a more specific approach at the local or hospital level. After the pandemic breakout, scientific societies continued to offer specific CO educational programs through web seminars or online competence courses.

In accordance with this educational purpose, ANMCO proposed specific pathways for the management of cardiac toxicity and focused booklets on controversial CO clinical issues. In view of the differences at the hospital level and the sometimes relevant regional distinctions (based on the fact that the Italian health system is regionally based), a single Italian pathway for CO cannot be drawn. A system too rigid could not be universally adopted, so we propose a basic outline to be adapted to local facilities and possibilities.

The cardiac side effects of anthacyclines and trastuzumab have been known since decades (13–15), and specific statements have long been available. At baseline, therapy and post-treatment evaluations are frequently performed in both general hospitals and cancer centers in Italy. In particular, we found a greater percentage of patients undergoing a post-treatment echocardiogram (68% as end-treatment and 45% at 1 year) in comparison with American administrative database evidence, showing that only 29.4% of American patients received an echocardiogram in the year following treatment (16). The relevance of classic and disease-specific cardiovascular risk factors has been clearly understood. A pretreatment check for cardiac toxicity risk factors is routinely performed. Recently, a joint paper from the Heart Failure Association of the European Society of Cardiology and the International Cardio-Oncology Society reaffirms the key role of pretreatment risk factor evaluation (17).

While survey respondents were confident in the clinical management of the cardiac side effects of anticancer drugs, the use of cardiac biomarkers and GLS techniques in monitoring cardiac toxicity is overall poor. The data offer a rather confused and uneven panorama regarding the behavior of different centers for the choice of different biomarkers, with a trend toward their increasing use in higher risk populations or in cases of suspected toxicity. Accordingly, the routine use of GLS seems to have increased over the past few years, but there is still a significant underutilization.

A growing problem in the daily practice of cardio-oncology is the management of cancer-related venous thromboembolism (VTE). The slight prevalence of cardiologists appears to be entrusted with handling VTE, and unfortunately, a multidisciplinary evaluation is rarely carried out. Study limitations should be taken into consideration. This analysis relies only on data from survey participants, which can in some cases be biased by their personal interpretation. The authors have not had direct access to Medical Center databases to check the accuracy of reported data. The general hospital category encompassed a wide range of hospital types, from small peripheral facilities to University hospitals. We did not report any subgroup analysis (e.g., imaging patterns, invasive procedures, and so on) based on hospital size because of both the lack of a clear definition of hospital category and the fact that these categories would be too small. Similarly, we did not run subgroup analysis comparing “general hospital” categories with cancer centers in any secondary item.

The picture of Italian CO, drawn by our survey, is a mixture of dark and light. Progress has undoubtedly been made over the last decade, but the challenges to face in the future are still numerous and complex. The role of national and international guidelines is now well-established, as is the management of older cardio-toxic drugs. Our results indicate two main objectives to be pursued to upgrade the clinical use of CO: (1) specific training provided locally by national scientific societies to both physicians and nurses and (2) closer collaboration at the single hospital level among specialists.

## Data Availability Statement

The datasets presented in this article are not readily available because participating centers do not provide authorization to share them. Requests to access the datasets should be directed to marialaura.canale@uslnordovest.toscana.it.

## Ethics Statement

The survey did not require approval by the Local Ethical Committee because it is based on physicians' opinions and administrative data only, without direct patient data collection.

## Author Contributions

MC, IP, CL, FC, DG, MG, NM, IB, and FT: study conception and design. MC, AC, IB, IP, CL, FT, SO, and GC: drafting of the manuscript or revising it critically for important intellectual content. MC, AC, FT, GR, SP, LT, IB, LR, GC, and SO: analysis and interpretation of data. All authors final approval of the manuscript.

## Conflict of Interest

The authors declare that the research was conducted in the absence of any commercial or financial relationships that could be construed as a potential conflict of interest.
