# Corrigendum: Portrait of Italian Cardio-Oncology: Results of a Nationwide Associazione Nazionale Medici Cardiologi Ospedalieri (ANMCO) Survey

**DOI:** 10.3389/fcvm.2022.920605

**Published:** 2022-05-10

**Authors:** Maria Laura Canale, Fabio Turazza, Chiara Lestuzzi, Iris Parrini, Andrea Camerini, Giulia Russo, Furio Colivicchi, Domenico Gabrielli, Michele Massimo Gulizia, Stefano Oliva, Luigi Tarantini, Nicola Maurea, Luigi Rigacci, Sandro Petrolati, Giancarlo Casolo, Irma Bisceglia

**Affiliations:** ^1^Division of Cardiology, Azienda USL Toscana Nord-Ovest, Versilia Hospital, Lido di Camaiore, Italy; ^2^Cardiology Unit, Fondazione IRCCS Istituto Nazionale dei Tumori, Milan, Italy; ^3^Cardiology Unit, Oncology Department, CRO National Cancer Institute, Aviano, Italy; ^4^Divisione di Cardiologia, Ospedale Mauriziano, Turin, Italy; ^5^Medical Oncology, Azienda USL Toscana Nord-Ovest, Versilia Hospital, Lido di Camaiore, Italy; ^6^SC Centro Cardiovascolare Ospedale Maggiore, Trieste, Italy; ^7^Division of Cardiology, San Filippo Neri Hospital, ASL Roma 1, Rome, Italy; ^8^Division of Cardiology, Azienda Ospedaliera San Camillo-Forlanini, Rome, Italy; ^9^Division of Cardiology, Garibaldi-Nesima Hospital, Catania, Italy; ^10^UOSD Cardiologia di Interesse Oncologico - IRCCS Istituto Tumori “GIOVANNI Paolo II” Bari, Bari, Italy; ^11^Division of Cardiology, Arcispedale S. Maria Nuova, Azienda USL – IRCCS di Reggio-Emilia, Reggio-Emilia, Italy; ^12^S.C. Cardiologia, Istituto Nazionale Tumori, IRCCS Fondazione G. Pascale, Napoli, Italy; ^13^UOC Ematologia, Azienda Ospedaliera San Camillo-Forlanini, Rome, Italy; ^14^Servizi Cardiologici Integrati, Azienda Ospedaliera San Camillo-Forlanini, Rome, Italy

**Keywords:** cardio-oncology, anthracyclines, trastuzumab, global longitudinal strain, cardiac biomarker, healthcare

In the original article, there were errors in “affiliations 10 and 11. Instead of “Division of Cardiology, Istituto Tumori Giovanni Paolo II, Bari, Italy,” it should be “UOSD Cardiologia di Interesse Oncologico - IRCCS Istituto Tumori “GIOVANNI Paolo II” Bari, Bari, Italy.”

Instead of “Divisione di Cardiologia, Arcispedale S. Maria Nuova, Reggio-Emilia, Italy,” it should be “Division of Cardiology, Arcispedale S. Maria Nuova, Azienda USL – IRCCS di Reggio-Emilia, Reggio-Emilia, Italy.”

The authors apologize for these errors and state that they do not change the scientific conclusions of the article in any way. The original article has been updated.

## Publisher's Note

All claims expressed in this article are solely those of the authors and do not necessarily represent those of their affiliated organizations, or those of the publisher, the editors and the reviewers. Any product that may be evaluated in this article, or claim that may be made by its manufacturer, is not guaranteed or endorsed by the publisher.

